# Acute paraumbilical vein recanalization: an unusual complication of acute pancreatitis

**DOI:** 10.1259/bjrcr.20150021

**Published:** 2015-04-20

**Authors:** R J Foster, G W Cowell

**Affiliations:** Victoria Infirmary, Glasgow, UK

## Abstract

Acute pancreatitis is associated with a number of well-known complications and imaging findings. Spontaneous recanalization of the paraumbilical veins as a consequence of pancreatitis in a patient with an otherwise normal liver is, however, a rare entity. This case report depicts this unusual complication as a consequence of gallstone pancreatitis in a patient with a non-cirrhotic liver and no clinical or radiological evidence of portal hypertension. There was recanalization of the paraumbilical veins followed by thrombosis, which is believed to have propagated in a retrograde fashion into distal branches of the otherwise patent portal vein. A literature search for similar cases such as this has yielded no results. Although rare, clinicians and radiologists alike need to be aware of this finding. This case discussion highlights the embryology and anatomy of the paraumbilical veins, as well as discusses the management of paraumbilical and portal vein thrombosis.

## Background

Acute pancreatitis has a number of well-recognized complications; however, recanalization of the paraumbilical vein is less well recognized and carries its own imaging findings and associated abnormalities. Although rare, clinicians and radiologists alike need to be aware of this finding.

## Case Presentation

A previously well 52-year-old male presented to the hospital with a 5-day history of abdominal tenderness, which was maximal in the left iliac fossa.

## Investigations

**Figure 1.**
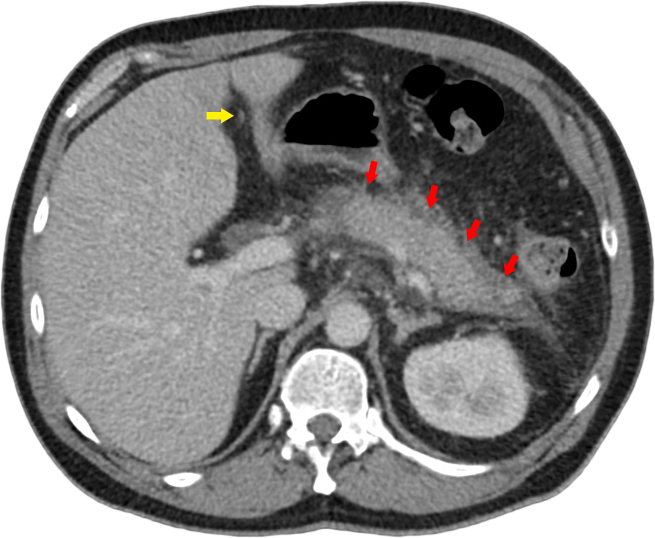
Intravenous contrast-enhanced (portal venous phase) multidetector CT axial image demonstrating a swollen oedematous pancreas (red arrows) with adjacent free fluid and fat stranding. Note is made of the normal and non-opacified paraumbilical vein (yellow arrow).

**Figure 2.**
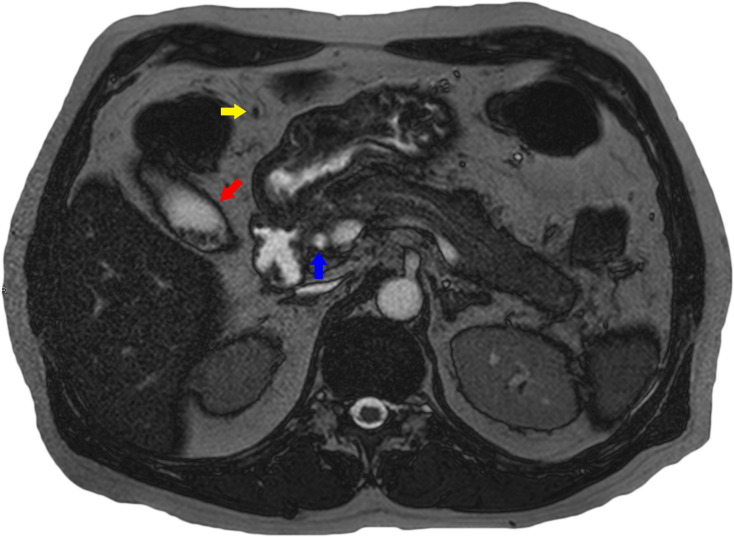
MRI transverse *T*_2_ weighted sequence demonstrating gallstones (red arrow), a normal calibre distal common bile duct with no internal filling defects/stones (blue arrow) and a normal non-dilated paraumbilical vein (yellow arrow).

An abdominal film performed in casualty was non-specific, while inflammatory markers were raised. Liver enzymes demonstrated a mild transaminitis and a normal amylase. The patient proceeded to get a CT scan that demonstrated a swollen, oedematous pancreas with peripancreatic inflammatory change (Figure 1). The gland enhanced uniformly and the portal vein was patent, in keeping with acute pancreatitis. Subsequent ultrasound of the abdomen revealed several small gallbladder calculi within an otherwise normal gallbladder and a normal calibre common bile duct. A subsequent magnetic resonance cholangiopancreatography (MRCP) study as an outpatient did not demonstrate choledocholithiasis (Figure 2).

**Figure 3.**
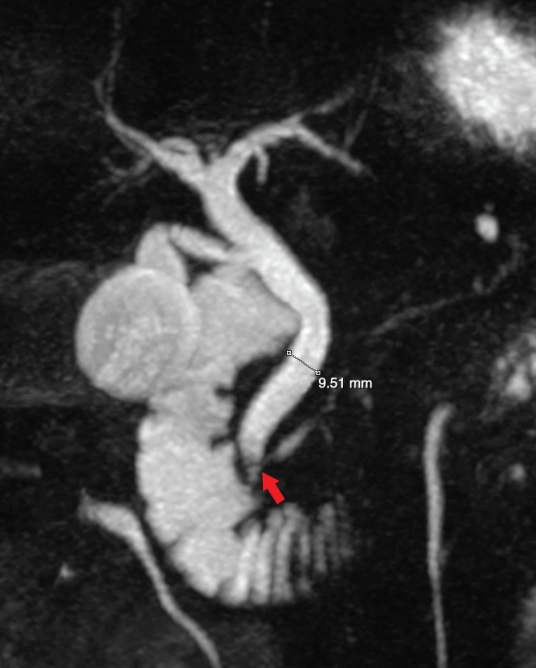
MRI *T*_2_ weighted maximum intensity projection three-dimensional reformat sequence depicting a newly dilated common bile duct ( >9 mm) and a small filling defect within the lower duct above the ampulla (red arrow).

**Figure 4.**
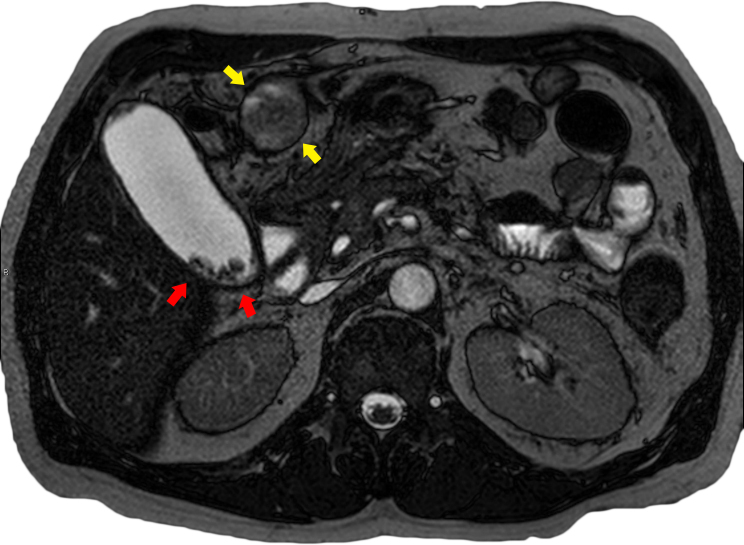
MRI *T*_2_ weighted transverse sequence again showing multiple small gallstones (red arrows). The paraumbilical vein is now significantly distended, measuring approximately 4 cm maximally (yellow arrows). Centrally, there is subtle high signal suggestive of slow flow with surrounding thrombus in varying stages of maturation and consequently of varying signal intensity. Surrounding the thrombosed vessel are significant inflammatory changes.

The patient re-presented several weeks later with recurrence of upper abdominal pain, vomiting, pale stools and dark urine. Inflammatory markers were mildly elevated but liver enzymes demonstrated a significant derangement with an obstructive pattern with mildly elevated bilirubin. Serum amylase at this point was abnormal (195 U l^−1^—normal reference range 0–100 U l^−1^). With a working diagnosis of obstructive ductal stones, a repeat MRCP was performed, showing a 12-mm calculus impacted at the ampulla (Figure 3). A further and incidental finding of acute recanalization of the paraumbilical vein was evident. There was a small central high signal indicative of flow with surrounding heterogeneous thrombus of varying degrees of maturation (Figure 4). Given this finding and the recent episode of acute pancreatitis, the concern was of portal vein thrombosis as a precipitant.

**Figure 5.**
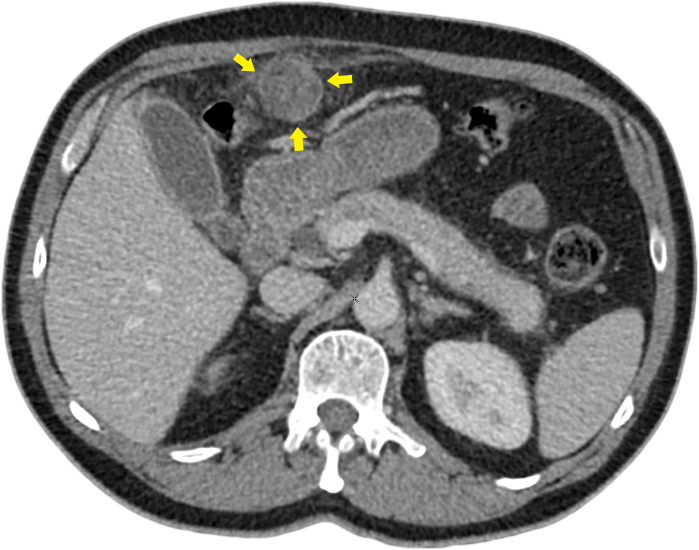
Intravenous contrast-enhanced (portal venous phase) multidetector CT axial image showing the thrombosed paraumbilical vein (yellow arrows), which measures 3.8 cm in maximal dimensions. No identifiable internal flow was evident either on the arterial phase or the portal venous phase.

**Figure 6.**
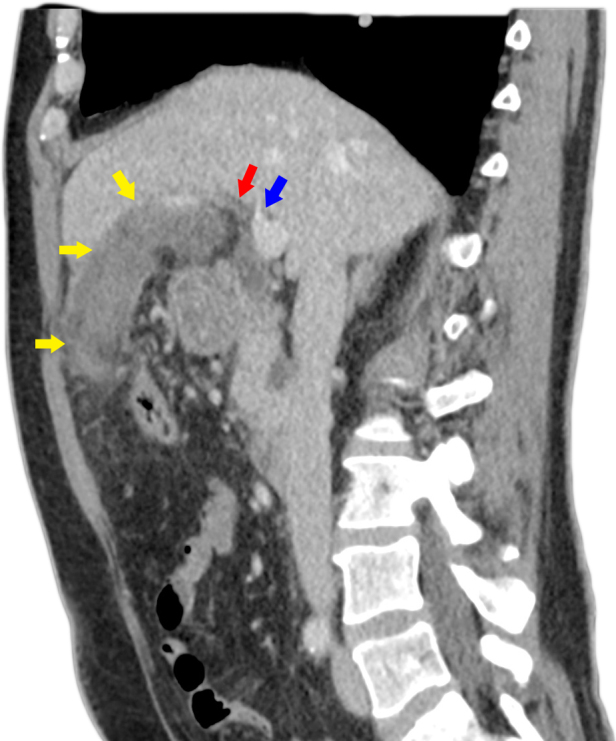
Intravenous contrast-enhanced (portal venous phase) multidetector CT sagittal image showing the thrombosed paraumbilical vein (yellow arrows) with thrombus extension into a branch of the left portal vein (red arrow; contrast opacified/non-thrombosed portal vein branch is illustrated by the blue arrow).

For further assessment, a triple-phase (non-enhanced, arterial and portal venous) contrast-enhanced CT scan of the liver was performed. The newly recanalized and partially thrombosed paraumbilical vein was shown to have a maximal cross-sectional diameter of 3.8 cm (Figure 5). Despite a marginal increase in attenuation between the arterial and portal venous phases, there was no discernible internal flow. Thrombus extended in a retrograde fashion to involve the left portal vein, with thrombosis in branches supplying segments II, III and IV (Figure 6) with corresponding relative arterial hyperenhancement in keeping with a transient hepatic attenuation difference (THAD). The remainder of the portal vein was patent, with no varices or cavernous transformation of the portal vein. The spleen was of normal size. The peripancreatic inflammation had resolved with a small residual pseudocyst.

The patient subsequently underwent endoscopic retrograde cholangiopancreatography, where the common bile ducts were noted to be moderately dilated. No ductal filling defect was present. Sphincterotomy and balloon sweep retrieved some stone debris.

## Treatment

The patient was anticoagulated and after discussion with haematology, 6 months of warfarin therapy was planned. Ultimately, it was planned that cholecystectomy would be the best course of definitive action.

## Discussion

Although portal vein thrombosis is well recognized following acute pancreatitis, acute recanalization of the paraumbilical vein is rare. The relatively small volume of portal vein thrombosis in this case is even more unusual, and demonstrates the need for clinicians and radiologists alike to be aware of uncommon complications of acute pancreatitis.

*In utero,* within the umbilical cord, a single or unpaired umbilical vein provides the foetus with oxygenated blood from the placenta. At the caudal rim of the navel, the unpaired umbilical cord connects with an intrafoetal left and right umbilical vein, which drain into the omphalomesenteric veins. The left and right umbilical veins are incorporated into the developing liver, establishing a connection with the capillary plexus. Consequently, there is communication with the sinus venosus, both directly and via hepatic anastomoses.^1^ The portal vein arises from segments of the vitelline veins, with the portal sinus derived from subhepatic intervitelline anastomosis connecting the umbilical vein—the predominant vessel of the foetal liver—to the portal system. The ductus venosus connects the portal sinus to the vena cava.^2^ With atrophy of the extrahepatic portion of the umbilical veins, blood from the umbilical cord reaches the sinus venosus and gets mixed with blood from the omphalomesenteric veins that pass through the liver.

Subsequently, there is atrophy of the right umbilical vein with the placental blood entering the liver via the left umbilical vein, which, following birth, is obliterated and the ductus venosus becomes the ligamentum venosum.^1^

Contrary to popular belief, the umbilical vein itself generally does not recanalize with portal hypertension. A study of patients with portal hypertension performed by Lafortune et al^3^ with correlation to post-mortem findings revealed no cases in which there was recanalization of the umbilical vein but instead showed an increase in the calibre and number of paraumbilical veins in patients with portal hypertension.

Recanalization of the paraumbilical veins is mainly secondary to portal hypertension, which is defined as elevation of hepatic venous pressure gradient of >5 mmHg. Portal hypertension is a consequence of increased hepatic vascular resistance that can broadly be classified as prehepatic, intrahepatic or posthepatic.

The prehepatic causes in an adult population are almost all as a result of portal vein thrombosis. Intrahepatic portal venous hypertension can be the result of cirrhosis, fibrosis and, less commonly, in non-cirrhotic patients owing to hepatosteatosis, schistosomiasis, tuberculosis etc. Posthepatic portal hypertension generally results from thrombosis of either the hepatic vein or inferior vena cava.

By far, the most common causes of portal venous hypertension are increased intrahepatic vascular resistance as a result of hepatic cirrhosis and a consequence of portal vein thrombosis.

The cause of portal vein thrombosis is often multifactorial but can be broadly classified into either local causes or systemic prothrombotic states. The presentation and clinical symptoms depend largely upon the speed of onset of thrombosis, ranging from acute infarction with subsequent haemorrhage in the acute setting to the sequelae of portal hypertension with variceal bleeding and cavernous transformation with multiple bridging collaterals bypassing the occlusion.^4,5^

Malignancy, in particular hepatocellular carcinoma (HCC), is an important local cause of portal vein thrombosis with pathological studies showing this tumour subtype having a greater propensity for venous invasion than do other primary hepatic tumours or metastatic deposits.^6^

Other local causes include trauma, surgery, intra-abdominal sepsis, splenectomy and regional inflammatory processes, such as pancreatitis.

Spontaneous recanalization with blood flow in the umbilical vein may occur during portal hypertension with the spontaneously reopened umbilical vein serving as a hepatofugal, decompressing collateral.^7^

The peculiarity of this case presentation lies in the fact that, between clinical presentations, there has been spontaneous recanalization of the paraumbilical veins and subsequently thrombosis in a patient with a non-cirrhotic liver and a largely patent portal venous system. A small volume of thrombus was noted within distal portal branches supplying the left lobe of the liver, which was most likely the precipitating cause. The remainder of the portal vein was patent.

The imaging findings in portal venous and paraumbilical vein thrombosis does vary depending on the chronicity of the clot.

On ultrasound, an acute thrombus typically appears either anechoic or hypoechoic and therefore is difficult to detect. With time and clot maturation, the thrombus becomes hyperechoic. Colour Doppler is useful in determining whether or not there is flow within the portal vein; however, in cases where flow is sluggish, discernible Doppler flow may be absent, giving false-positive results.

CT scan appearance of thrombus again varies with the age of the clot. On unenhanced studies, an acute clot can appear relatively hyperdense relative to the adjacent liver parenchyma, becoming iso-hypodense over time. With the administration of iodinated contrast, a clot appears as a filling defect, either partial or complete.^8^

Because the liver has a dual blood supply from both the hepatic artery and the portal vein, physiological changes can affect the enhancement pattern. One such phenomenon is known as THAD on CT or transient hepatic intensity difference on MRI, which equates to relative hyperenhancement of a liver territory during the hepatic arterial phase. There are a number of different causes that include lesions, such as HCC, and other non-lesional causes, such as portal venous thrombus. The mechanism is thought to be secondary to a relative mismatch in flow between the hepatic artery and the portal venous supply, with a relative increase from the hepatic artery.^9,10^

MRI has been stated to be the most sensitive imaging modality for the detection of portal vein thrombosis with a sensitivity of 98% and a specificity of 99%; however, it does have limitations to its use, especially in acutely unwell patients. Portal venous thrombus has a varied appearance on MRI, which again is related to age of the thrombus and the specific MRI sequence. Classically, in the acute setting, a clot will appear as a high signal intensity on both *T*_1_ weighted and *T*_2_ weighted sequences. With increasing chronicity, there is loss of *T*_2_ weighted signal. MR angiography with the administration of gadolinium, similar to CT, is able to demonstrate filling defects as a result of clot. Tumour thrombus generally enhances, is typically hyperintense on *T*_2_ weighted sequences and is an important differential for portal vein thrombosis.^11^

The second admission with obstructing distal stones was the cause of the patient’s abnormal liver enzyme tests. It is difficult to definitively determine whether it was the distal ductal stone or the acute thrombosis of para-umbilical veins that was the cause of the patient's abdominal pain or possibly a combination of both. Recanalization of the umbilical vein in the absence of cirrhosis and significant portal vein thrombosis is extremely rare, and this case serves as a reminder for the less common causes of right upper quadrant pain and complication of acute pancreatitis.

## Learning Points

1. Recanalization of the paraumbilical vein is an important but rare consequence of acute pancreatitis.
2. Portal vein thrombosis usually accompanies this finding, and as this case demonstrates, thrombus load can vary.
3. The reporting radiologists need to be vigilant to such complications at imaging, particularly as studies, such as MRCP, are optimized to detect portal vein thrombosis or paraumbilical vein recanalization.
